# Disease control of acromegaly does not prevent excess mortality in the long term: results of a nationwide survey in Italy

**DOI:** 10.1007/s40618-023-02257-3

**Published:** 2024-01-12

**Authors:** M. Arosio, V. Sciannameo, A. Contarino, P. Berchialla, S. Puglisi, A. C. Pesatori, E. Ferrante, M. Filopanti, R. Pivonello, F. Dassie, V. Rochira, S. Cannavò, E. De Menis, F. Pigliaru, S. Grottoli, V. Cambria, M. Faustini-Fustini, M. Montini, A. Peri, F. Ceccato, E. Puxeddu, G. Borretta, M. Bondanelli, D. Ferone, A. Colao, M. Terzolo, G. Reimondo

**Affiliations:** 1 Endocrinology Unit, Fondazione IRCCS Ca’ Granda Ospedale Maggiore Policlinico, Via Francesco Sforza 35, 20122 Milan, Italy; 2 Department of Clinical Sciences and Community Health, University of Milan, Milan, Italy; 3 Statistical Unit, Department of Clinical and Biological Sciences, San Luigi Gonzaga Hospital, University of Turin, Orbassano, Italy; 4 Internal Medicine, Department of Clinical and Biological Sciences, S. Luigi Hospital, University of Turin, Turin, Italy; 5 EPIGET LAB, Department of Clinical Sciences and Community Health, University of Milan, Milan, Italy; 6 Epidemiology Unit, Fondazione IRCCS Ca’ Granda Ospedale Maggiore Policlinico, Milan, Italy; 7 Division of Endocrinology, Department of Clinical Medicine and Surgery, University Federico II Di Napoli, Naples, Italy; 8 Internal Medicine, Department of Medicine, DIMED, University of Padova, Padua, Italy; 9 Endocrinology Unit, Department of Biomedical, Metabolic and Neural Sciences, University of Modena and Reggio Emilia, Modena, Italy; 10 Endocrinology Unit, Department of Medical Specialties, Azienda Ospedaliero-Universitaria of Modena, 41126 Modena, Italy; 11 Department of Human Pathology “G. Barresi”, University of Messina, Messina, Italy; 12 Internal Medicine 2-Endocrine-Metabolic Department, Treviso Hospital, Montebelluna, Treviso, Italy; 13 Endocrinology Unit, AOU Cagliari, Cagliari, Italy; 14 Division of Endocrinology, Diabetes and Metabolism, Department of Medical Science, University of Turin, AOU Città della Salute e della Scienza, Turin, Italy; 15 IRCCS Institute of Neurological Sciences of Bologna, Bologna, Italy; 16 Ambulatori di Endocrinologia, Humanitas Gavazzeni, Bergamo, Italy; 17 Pituitary Diseases and Sodium Alterations Unit, Endocrinology, Department of Experimental and Clinical Biomedical Sciences “Mario Serio”, Careggi University Hospital, University of Florence, Florence, Italy; 18 Department of Medicine (DIMED), University of Padova, Padua, Italy; 19 Endocrinology Unit, Padova University Hospital, Padua, Italy; 20 Department of Medicine and Surgery, Section of Internal Medicine and Endocrine and Metabolic Sciences, University of Perugia, Perugia, Italy; 21 Division of Endocrinology and Metabolism, Santa Croce and Carle Hospital, Cuneo, Italy; 22 Section of Endocrinology, Geriatrics and Internal Medicine, Department of Medical Sciences, University of Ferrara, Ferrara, Italy; 23 Endocrinology Unit, Department of Internal Medicine and Medical Specialties (DI.M.I.), University of Genoa, Genoa, Italy; 24 Endocrinology Unit, IRCCS Ospedale Policlinico San Martino, Genoa, Italy

**Keywords:** Acromegaly, Mortality, IGF1, Therapy, Epidemiology

## Abstract

**Objective:**

This study aimed to assess the long-term outcome of patients with acromegaly.

**Design:**

This is a multicenter, retrospective, observational study which extends the mean observation period of a previously reported cohort of Italian patients with acromegaly to 15 years of follow-up.

**Methods:**

Only patients from the centers that provided information on the life status of at least 95% of their original cohorts were included. Life status information was collected either from clinical records or from the municipal registry offices. Standardized mortality ratios (SMRs) were computed comparing data with those of the general Italian population.

**Results:**

A total of 811 patients were included. There were 153 deaths, with 90 expected and an SMR of 1.7 (95% CI 1.4–2.0, *p* < 0.001). Death occurred after a median of 15 (women) or 16 (men) years from the diagnosis, without gender differences. Mortality remained elevated in the patients with control of disease (SMR 1.3, 95% CI 1.1–1.6). In the multivariable analysis, only older age and high IGF1 concentrations at last available follow-up visit were predictors of mortality. The oncological causes of death outweighed the cardiovascular ones, bordering on statistical significance with respect to the general population.

**Conclusions:**

Mortality remains significantly high in patients with acromegaly, irrespectively of disease status, as long as the follow-up is sufficiently long with a low rate of patients lost to follow-up. Therapy strategy including radiotherapy does not have an impact on mortality. Oncological causes of death currently outweigh the cardiovascular causes.

**Supplementary Information:**

The online version contains supplementary material available at 10.1007/s40618-023-02257-3.

## Introduction

Acromegaly is a rare endocrine disease resulting from chronic exposure to excess GH and IGF1, mostly due to a GH-secreting pituitary adenoma [1, 2], with an estimated prevalence ranging from 28 to 137 cases per million inhabitants [3]. Acromegaly carries a heavy [4] disease burden, including an increased risk of cardiovascular, metabolic, and neoplastic diseases, which results in excess mortality with an estimated loss of life expectancy of 10 years and a mortality 2–3 times higher than the general population [5–8]. In most studies published in the last decade, life expectancy was found to be improved in adequately treated acromegalic patients, with a similar standardized mortality ratio (SMR) to the general population [9–14], particularly in patients with controlled disease [15]. The availability of new medical treatments for acromegaly and its comorbidities, along with improvements in pituitary surgery and radiotherapy, leading to a reduced occurrence of iatrogenic hypopituitarism, may explain this reduction in mortality [16–18].

The leading causes of death have also changed over time with a reduction in cardiovascular deaths and an increase in oncological deaths [4, 10, 12, 13, 16–18]. Unexpectedly, a recent nationwide Finnish study found that all-cause mortality was greater in acromegalic patients than in the general population [19]. The increase in mortality was observed only after a 20-year follow-up and not in a previous study on the same population with a 12-year follow-up [9]. This suggests that an extended observation may be necessary to detect differences in life expectancy with the general population, given the relatively young age of acromegalic patients at diagnosis [18].

In 2012, we published an Italian nationwide observational study on 1512 acromegalic patients with a mean follow-up of 10 years [10]. In that study, we observed that mortality in the overall cohort was similar to the general population, while being significantly higher in the subgroup of patients with active disease.

The present survey aims to assess the long-term mortality in the same cohort of acromegalic patients (i.e., the 2012 cohort), and to identify the predicting factors and the specific causes of death.

## Methods

This is a multicenter, retrospective, observational study, which extends the observation period of a previously reported cohort of Italian patients with acromegaly [10, 20]. Eighteen out of the 24 tertiary referral centers that participated in the previous study sent follow-up data for a total of 1033 patients representing 88% of the original cohort of 1178 patients. The database was closed for analysis in December 2014.

The life status information and the causes of death were collected from clinical records of 720 patients and from the municipal registry offices of the remaining 313 patients. In order to reduce the number of patients lost to follow-up, only the 11 centers that provided life status information of at least 95% of their original series contributed to the main cohort, which included 811 patients.

Details on inclusion and exclusion criteria have been reported previously [10, 20]. The main inclusion criteria were age at diagnosis > 18 years, Italian residency, and diagnosis of acromegaly made between 1 January 1980 and 31 December 2002. Patients with GH hypersecretion due to ectopic GHRH secretion and known family history of pituitary adenoma were excluded. The data were collected retrospectively using a computerized dataform developed using Microsoft Access 2000 and then transferred to Microsoft Excel for the analysis.

The present survey gathered additional information on novel therapies for acromegaly, anterior pituitary deficiencies and treatment, GH and IGF1 levels at last follow-up, cardiovascular, cerebrovascular, and oncological morbidities, and mortality. At their last available follow-up visit, patients were considered to have controlled disease if their IGF1 blood concentrations were below the upper limit of age- and sex-adjusted normal range, or active disease if their IGF1 were high. It should be emphasized that the introduction and the increasing use of GH-antagonists mean that only IGF1 can be used to evaluate disease control [15, 16]. IGF1 concentrations were expressed either in absolute values or as ULN (concentration of the patient/upper normal age limit and sex-adjusted for the specific laboratory).

Diagnosis of anterior pituitary deficiencies was performed according to international guidelines [21] by a complete hormonal evaluation including the use of stimulation tests when indicated. The presence of comorbidities and risk factors for mortality was evaluated at the time of the last follow-up visit and defined according to accepted international criteria [22, 23]. The causes of death were obtained from death certificates or medical records.

For statistical analysis, categorical variables are summarized as numbers and percentages, while continuous variables as mean and SD, or median and IQR. Categorical variables were compared using the *χ*^2^. Depending on the data distribution, the paired *t*-test or the Wilcoxon signed-rank test was carried out. Predictors of mortality were evaluated via a multivariate proportional hazards Cox regression model. A multivariate model was developed based on clinical discussions and statistical selection procedures. Model selection was performed using an automatic approach based on the Akaike Information Criteria (AIC) method. Given the large number of covariates, a genetic algorithm was employed to explore the candidate set of models. Model goodness of fit was computed with reference to the Brier score (the closer to 0, the better). The results are presented as hazard ratios (HRs) and 95% confidence intervals (95% CI). The Schoenfeld residuals were visually inspected to assess violations of the proportional hazards assumption.

Standardized mortality ratios (SMRs) with 95%CI were computed as the ratio between observed and expected death calculated by multiplying the age, sex, and calendar-year mortality rate in Italy by the number of person-years. Mortality rates of the population in Italy were retrieved from the Italian National Institute of Statistics. The significance level was set at *p* < 0.05. All statistical analyses were performed using R v. 4.0.0

## Results

### Characteristics of the patient cohort

The main cohort included 811 patients, 474 F (58.4%) and 337 M (41.6%), with a mean age at the last follow-up or death of 60.2 ± 13.7 years. The total mean follow-up was 15.7 ± 8.3 years (median: 15.6, IQR 10–21). The main demographic and clinical characteristics of the patients are reported in Table 1. These were similar to those of the entire group of 1033 patients for which we received information (Supplementary Table 1). As we already showed in our previous study on the mortality of acromegaly [10], estimated duration of the disease prior to diagnosis was 74 months (median: 60 months; IQR 36–96), without significant differences between the two genders.

**Table 1:** The study cohort (*n* = 811)

Parameter	Value	Missing data (%)
Age at diagnosis, mean ± SD, years	44.7 ± 13	0
Follow- up, mean ± SD, years	15.7 ± 8.3	0
GH at diagnosis, mean ± SD, μg/dL	34.8 ± 70	6.0
GH at last visit, mean ± SD, μg/dL	2.4 ± 4.9	19.6
IGF1 at diagnosis, mean ± SD, μg/dL ULN	760 ± 338 3.85 ± 4.2	28.5
IGF1 at last visit, mean ± SD, μg/dL ULN	209 ± 152 0.8 ± 0.5	10.5
Patients with controlled disease at last visit	558 (76.8)	10.5
Hypothyroidism, *n* (%)	222 (30)	8.8
Hypoadrenalism, *n* (%)	157 (21)	8.6
Hypogonadism, *n* (%)	242 (34)	9.1
GH deficiency, *n* (%)	59 (8.4)	12.9

GH, growth hormone; IGF1, insulin growth factor I; SD, standard deviation; ULN, upper limits of normal

At the last follow-up, disease status was known in 726 patients (89.5%). High age-adjusted serum IGF1 concentrations were still observed in 168 patients (23.1%), while 558 patients were considered as controlled (527 with IGF1 within the normal range and 31 below), of which 312 (56%) were considered as cured, since they were untreated at the last follow-up. The patients with controlled disease were slightly but significantly older (61.6 ± 13 vs 58 ± 14 years, *p* < 0.005).

Anterior pituitary deficiencies varied from 8.4% for GH deficit to 34% for hypogonadism, as shown in Table 1. At the last follow-up, the prevalence of diabetes mellitus was 28%, hypertension 53%, obesity 29%, OSAS 11%, and 22% of the patients were smokers. The frequency of both diabetes and hypertension was not significantly different between patients with active or controlled disease (diabetes: 27.5% vs 31.6%, hypertension: 56.3% vs 48.7%, respectively).

During the follow-up, 9.8% of patients developed cardiovascular diseases: 6.1% had myocardial infarction, and 3.7% developed heart failure. Occurrence of malignancy was reported in 17%.

### Treatments

Data on treatment of acromegaly were reported in 93% of patients. Of these, 648 had undergone one or more pituitary surgeries within a mean of 11 ± 5 years before the last observation, and 226 had received radiotherapy (164 conventional radiotherapy, 59 radiosurgery, 3 both). Furthermore, 607 patients had received one or more lines of medical therapy (somatostatin analogs in 569 patients, pegvisomant in 103, and dopamine agonists in 242). Medical treatments were given alone or in various combinations. A total of 120 patients were treated by surgery alone, 108 by medical therapy alone (mostly with somatostatin analogs), and 5 by radiotherapy alone. Additionally, 286 patients were treated with a combination of surgery and pharmacotherapy without radiotherapy, and 5 patients did not receive any form of treatment. Surprisingly, 61 patients with uncontrolled disease were not receiving any medical treatments at the last follow-up.

### Mortality

By the end of December 2014, 153 deaths (19%) had been observed, while 90 were expected with a SMR of 1.7 (95% CI 1.4–2.0, *p* < 0.001) (Table 2). Mean age at death was slightly higher in women than in men (70 ± 11 years vs 66 ± 12 years, *p* = NS). Death occurred after a median of 16 years (IQR 9–23) from the diagnosis of acromegaly in women, and after a median of 15 years (IQR 9–21) in men. The SMR was significantly higher both in men (1.96, 95% CI 1.5–2.5) and women (1.49, 95% CI 1.2–1.8), without a statistically significant gender difference.

**Table 2:** Standardized mortality rates (SMR) in the whole cohort, and in groups of patients with different disease status at last follow-up, in comparison to the general population in Italy

	*N*. of patients (%)	*N*. observed deaths (%)	*N*. expected deaths	SMR [95% CI]
Total cohort	811	153 (19)	90.0	1.7 [1.4–2.0]
Controlled disease	558 (69)	88 (16)	66.0	1.3 [1.1–1.6]
Active disease	168 (21)	43 (26)	17	2.5 [1.8–3.3]
Unknown	85 (10)	22 (26)	7.0	3.2 [2.0–4.7]

Mortality remained significantly higher than expected in the Italian population, also in patients with controlled disease (SMR 1.3, 95% CI 1.1–1.6). However, the SMR was significantly lower than in patients with active disease or patients lost to follow-up (Table 2). Among 61 patients with uncontrolled disease and without any medical therapy at the last follow-up, only 2 patients had never undergone any therapy for acromegaly. The remaining 59 patients underwent prior medical therapy (*n* = 44) or surgery (*n* = 50) or radiotherapy (*n* = 21) or variable combinations of these throughout their disease history.

In the overall cohort, several factors were significantly associated with increased mortality, such as advancing age, lack of normalization of IGF1, higher IGF1 at diagnosis, higher GH concentrations at last control, the presence of diabetes mellitus, hypertension, cardiovascular events, and the absence of malignancy. However, the prevalence of obstructive sleep apnea syndrome (OSAS), smoking, and obesity did not significantly differ such as the presence of anterior pituitary deficiencies (Table 3).

**Table 3:** Comparison of biochemical characteristics, comorbidities, and treatment of patients who were alive or deceased at the last follow-up

	Alive	Dead	*p* value
N. of patients	658	153	
Sex, M (%)	277 (42.1)	60 (39.2)	0.575
Age at last visit or death, mean ± SD	58 ± 12	70.3 ± 11	< 0.01
Follow-up, median [95% CI]	15.6 [11–21]	15.2 [9–22]	NS
IGF1 normalization, *n* (%)	520 (79)	103 (67.2)	0.013
IGF1 at diagnosis (ULN), mean ± SD	3.5 ± 3.1	6.5 ± 8.1	< 0.001
IGF1 at last FU (ULN), mean ± SD	0.83 ± 0.45	0.85 ± 0.58	0.701
GH at diagnosis μg/L, mean ± SD	33.9 ± 64.8	38.8 ± 89.3	0.451
GH at last FU μg/L, mean ± SD	2.2 ± 4.5	3.4 ± 6.2	0.009
*Pituitary deficiencies*			
GH deficiency, *n* (%)	44 (7.6)	15 (12)	0.149
Central hypothyroidism, *n* (%)	183 (30)	39 (29.8)	1.000
Central hypoadrenalism, *n* (%)	130 (21.2)	27 (20.9)	1.000
Central hypogonadism, *n* (%)	199 (33.6)	43 (33.6)	1.000
*Comorbidities and risk factors*			
Diabetes mellitus, *n* (%)	158 (26)	50 (38.2)	0.006
Hypertension, *n* (%)	300 (49.6)	91 (72.8)	< 0.001
OSAS, *n* (%)	43 (9.7)	12 (17.1)	0.094
Coronary artery disease, *n* (%)	19 (3.4)	21 (20.2)	< 0.001
Heart failure, *n* (%)	17 (3.0)	8 (7.5)	0.047
Smoking habit, *n* (%)	90 (21)	27 (28.1)	0.170
Absence of malignancy, *n* (%)	528 (80.2)	81 (52.9)	< 0.001
*Type of therapy*			
Pituitary surgery, *n* (%)	542 (82)	106 (69)	< 0.001
Conventional RT, *n* (%)	129 (20.9)	38 (28.1)	0.087
Radiosurgery, *n* (%)	56 (8.9)	6 (4.3)	0.108
Pegvisomant, *n* (%)	95 (15)	8 (6)	0.006
SSA, *n* (%)	476 (75.8)	93 (68.4)	0.091
Dopaminergic, *n* (%)	196 (31.6)	46 (33.8)	0.690

FU, follow-up; GH, growth hormone; IGF1, insulin growth factor I; NS, not significant; OSAS, obstructive sleep apnea syndrome; RT, radiotherapy; SSA, somatostatin analogs; ULN, upper limits of normal

Interestingly, patients who were still alive had undergone pituitary surgery more frequently than those patients who had died (82% vs 69%, *p* < 0.001) or who had been given medical therapy with pegvisomant (15% vs 6%, *p* < 0.01) alone or in combination. The impact of radiotherapy on mortality was not significant.

At the multivariable regression analysis, older age and active disease were the only positive predictors of mortality, irrespectively of the type of treatment (Table 4). We did not identify any statistically significant association between the mean time delay for diagnosis and mortality in acromegaly (HR = 1.17, 95% CI 0.833–1.64, *p* = 0.366). The addition in the statistical model of radiotherapy, hypertension, and/or diabetes did not change the results. (Supplementary Table 2).

**Table 4:** Multivariable model for independent predictors of mortality (*n* = 612)

Variables	HR	95% CI	*p* value
Gender	1.06	0.70–1.60	0.787
Age	3.92	2.75–5.58	< 0.001
IGF1 above ULN at last follow-up	2.63	1.71–4.04	< 0.001
*Therapy*			
Surgical vs multimodal	1.53	0.80–2.93	0.204
Medical vs multimodal	1.01	0.59–1.74	0.968

HR, hazard ratio; IGF1, insulin growth factor I; multimodal therapy includes any combination of at least two types of treatment (medical, surgical or radiation, either radiotherapy or radiosurgery); ULN, upper limits of normal

The causes of death were known in 109 patients (71.2%) (Table 5). The main cause was cancer (48 patients, 44%), followed by cardiovascular diseases (44 cases, 40%). The most frequent diagnosis in patients who died of cancer were as follows: colon and pancreas (7 cases each) followed by breast (6), lung (5), bladder (4), and gastric cancer (4). The time between the diagnosis of acromegaly and the identification of the tumor was available only for 26 of the 48 patients who died from cancer, with a mean of 13.3 years (standard deviation of 7.97 years) and a median of 11.6 years (interquartile range 8.3–19.6 years). Among patients dying of vascular diseases, 17 patients had heart failure (16%), 13 stroke (12%), 12 myocardial infarction (11%), and two sudden death. Regarding the 6 patients (5.5%) who died from respiratory diseases, the reported cause of death was respiratory failure and pneumonia. However, it is likely that the frequency of OSAS has been underestimated both in the entire cohort and in the subgroup of deceased acromegalic patients.

**Table 5:** Known causes of death, with standardized mortality rate (SMR), in deceased acromegalic patients compared to the general Italian population of the same age and sex died in the same period

Causes of death	*N* (%)	SMR (95% CI)
Cancer	48 (44)	1.32 [0.99–1.75]
Cardiovascular and cerebro-vascular diseases	44 (40)	
Coronary artery disease	29	0.72 [0.49–1.03]
Stroke	13	1.59 [0.89–2.65]
Sudden cardiac death	2	
Respiratory diseases	6 (6)	1.23 (0.5–2.55)
Others	11 (10)	

The distribution of the leading causes of mortality did not significantly differ from that of the general Italian population matched for age and sex, except for cancer, which bordered on significance (Table 5).

## Discussion

This survey of more than 800 acromegalic patients in Italy, with a follow-up of 15 years, shows that even with adequate treatment and IGF1 normalization, the increased mortality risk can be reduced, but not completely reversed. This finding is in contrast with most available evidence [4]. The seminal meta-analysis by Holdaway and colleagues found that patients with a normal IGF1 had a mortality close to that of the general population, unlike patients in whom the IGF1 remained elevated [7]. Holdaway's findings have then been later confirmed in most studies [4, 11–13, 15], including our previous survey [10] with a shorter follow-up of 10 years.

The key factor seems to be the much longer follow-up period adopted in our current study, which highlights the true extent of the residual excess mortality in patients with acromegaly, a disease in which the diagnosis is on average when the patient is 45 years old. It is important to note that this occurs despite the attainment of the hormonal control in a number of patients that increased over time. This results from our study of 811 patients thus confirm what was found in a cohort of 333 patients in Finland [19].

Even more important is the active search of the life status of more than 300 patients lost to follow-up in order to prevent underestimating the death rates [18]. By including only the centers able to provide information on the life status of more than 95% of the patients originally included in our first study, we were able to reduce the number of patients lost to follow-up to just 3.7%. In the French study ACROSPECT, 362 patients were tracked out of more than 500 lost to follow-up in 25 tertiary Endocrinology Centers, of which 62 had died (17%) without the center being aware [24].

The SMR of the patients lost to follow-up was also extremely high in the Swedish Pituitary Register: three times higher than in the remaining acromegalic population [15]. Whatever the possible cause, the fact remains that since the patients lost to follow-up have the greatest risk of death, having the most comprehensive follow-up information is key to an accurate estimate of the death rate.

We did not observe any significant gender differences in the mortality rate compared to the general age-matched population. There are controversial data on this topic as discussed in a recent review [25]. In fact, studies carried out in Finland [19] and Korea [26] support a gender difference in mortality. The Korean study included 718 patients and found that mortality was higher in a statistically significant way only for women (SMR 1.75, 95% CI 1.07–2.84, vs 1.51, CI 0.83–2.78 in men). However, in both the Finnish and Korean studies the comparison was made with a control group and not with the general nationwide population. In agreement with the review cited above [25], we can conclude that although there is no evidence of a clear gender influence on mortality, women with acromegaly seem to lose the advantage derived from gender, as in other chronic diseases.

One point that seems to be highlighted in all studies is that the age of men at diagnosis is lower than that of women [15, 25, 27]. In our series, the difference was about 4 years (43 vs 47 years), and this difference was reflected in a 4-year lower mean age at death in men than in women (66 vs 70 years) after a median of 15 years for men and 16 years for women from diagnosis. Not surprisingly, and in agreement with all the studies, old age remains the main predictor of mortality along with the disease is controlled or not [4, 10–15, 19]. Conversely, we did not identify any statistically significant association between diagnostic delay and increased mortality in acromegaly, confirming what we observed in our previous study [10].

As for causes of death, diseases of the circulatory system and tumors were the two most frequent and were responsible for 8 out of 10 deaths of the acromegalic patients. We found a greater number of oncological causes compared with the Italian population, and this number was very close to significance, while stroke and cardiovascular death showed a similar HR as the general population. Thus, we confirm and extend the trend in recent years [4, 12, 13, 16, 18, 28] showing an increase in oncological causes of death over cardiovascular ones, at variance with the traditional observation of cardiovascular mortality as the primary cause in acromegaly. Unfortunately, we did not have the necessary information to evaluate whether the tumor was diagnosed during the active or remission phase of acromegaly.

The relationship between GH excess and IGF1 and the onset of cancer has always attracted much interest, although it is difficult to demonstrate a clear cause and effect [4, 29–31]. In agreement with some studies [14, 20, 26, 28, 32, 33], we observed a higher incidence of cancer in the acromegalic population, and this finding is also in accordance with a meta-analysis [34]. However, there are studies at variance [8, 30, 31, 35], including the German and French Registries [13, 36].

In any case, it is tempting to hypothesize that life-long exposure to GH and IGF1 excess may favor the progression of a malignant tumor leading to death many years later, often when the acromegalic disease is under control [4, 30, 37]. It is worth noting that in the Finnish study the oncological causes of death increased in the acromegalic but not in the control population starting from 10 years after diagnosis [19]. In many studies, the impact of the hormonal excess was likely also mitigated by the high level of clinical surveillance and excellence treatments provided to patients managed at tertiary centers. In view of the limited numbers, it is not possible to understand whether a particular type of tumor is more closely associated with acromegaly. In our series the most prevalent tumors were colon, lung, breast, and pancreas, the same sites observed in the Finnish study [19], all tissues with a high expression of GH/IGF1 and their related receptors [4].

As for comorbidities, the frequency of diabetes mellitus and hypertension were 28% 53%, respectively, at the last visit. These figures are in line with those of the literature [4, 18]. The frequency of these comorbidities is higher than at diagnosis of acromegaly, reflecting the fact these comorbidities become more frequent with age, both in the acromegalic and in the general population [10, 18]. The prevalence of diabetes mellitus and hypertension was significantly higher among patients who had died than among those still alive. These findings were expected [8], and in the ACROSTUDY [38], which included 2090 acromegalic patients treated with pegvisomant, patients with hypertension had a three-fold higher mortality compared with normotensive patients. In addition, a recent study from the Swedish Register, comparing 254 acromegalic patients affected by diabetes and 532 unaffected, showed a hazard ratio of 1.58 for overall mortality in the diabetic group [39].

There are many confounding factors to consider when trying to understand the impact of these comorbidities on mortality, as they are associated with the severity of acromegaly, and glycemic control may be affected by the type of acromegaly treatment. Furthermore, diabetes and hypertension are closely related to each other and contribute to determining the patient's cardiovascular risk; consequently, controlling diabetes and hypertension is fundamental. Given the close link between hypertension and diabetes with age and a potential immortal bias [40], this may have contributed to the fact that in our multivariable analysis neither of these two comorbidities significantly contributed to mortality.

In the recent Korean nationwide cohort study mentioned above, not only was the risk of all-cause death significantly higher in the acromegalic population than in controls of same age and gender, but the difference remained significant also after adjusting for type 2 diabetes, hypertension, dyslipidemia, and other risk factors [41]. This suggests that the direct effect of GH/IGF-1 excess on mortality is greater than that of comorbidities.

In our study, it is also difficult to evaluate the impact of the different kinds of therapy since they were introduced at different time and there was also a potential immortal time bias [40]. This is particularly true for pegvisomant, which only became a treatment for acromegaly in the early 2000s.

Our multivariable analysis highlights that there is no clear superiority of any treatment with respect to survival. The impact on survival depends on whether or not hormone control is achieved, and not on the type of treatment.

These results agree with those of other studies [43] that also did not record a significant impact of a given treatment strategy on mortality risk. It is noted that in our study radiotherapy, which is traditionally associated with an increased risk of mortality [4, 7, 8, 10, 19, 42] despite a good efficacy in normalizing hormonal hypersecretion [44], lost its negative impact on long-term mortality. This finding is in agreement with other recent observations [15].

The strength of the present study is the inclusion of a large number of patients with disease control, the long duration of follow-up, and the low number of patients lost to follow-up. Limitations include the retrospective nature of the study with impossibility to evaluate the cumulative exposition to excess GH and IGF1, the uncertain applicability of results to patients diagnosed after 2002, the unknown cause of death in many patients, and the lack of information about the severity of comorbidities.

In conclusion, we have shown that after a long-term follow-up, mortality in both men and women with acromegaly remains significantly higher than in the general population, even in the large group of disease-controlled patients surveyed in this study. The choice of the therapeutic strategy does not seem to have a major impact on long-term mortality.

### Supplementary Information

Below is the link to the electronic supplementary material.

Supplementary file1 (DOCX 19 KB)
