# Supplementary material for: Disease control of acromegaly does not prevent excess mortality in the long term: results of a nationwide survey in Italy
Source: J Endocrinol Invest. 2024 Jan 12;47(6):1457–65. doi: 10.1007/s40618-023-02257-3 (PMC11142937; doi:10.1007/s40618-023-02257-3)
Supplement: Supplementary file 1 — Supplementary file1 (DOCX 19 KB) [file 40618_2023_2257_MOESM1_ESM.docx]

**Supplementary Table 1.** Descriptive characteristics of the 1033 acromegalic patients with available follow-up data.

|  | **Overall** | **Study cohort** | **P value** |
| --- | --- | --- | --- |
| N. of centers | 18 | 11 |  |
| N. of patients reported/total, n (%) | 1033/1178 (88) | 811/842 (96) |  |
| Gender, M (%) | 427 (41.3) | 337 (41.6) | 0.3 |
| Age at diagnosis, mean ± SD, y | 44.7 ± 13 | 44.7 ± 13 | 0.9 |
| Age at death or at last visit, mean ± SD, y | 60.8 (13.7) | 60.2 ± 13.7 | 0.9 |
| Follow- up, mean ± SD, y | 15.5 (8.3) | 15.4 ± 8.3 | 0.8 |
| Smoking habit, n (%) | 134 (20.7) | 117 (22.3) | 0.5 |
| Obesity, n (%) | 185 (26.9) | 156 (28.8) | 0.5 |
| Hypertension, n (%) | 492 (54.5) | 391 (53.6) | 0.7 |
| Diabetes, n (%) | 250 (26.9) | 208 (28) | 0.6 |
| GH deficiency, n (%) | 70 (7.8) | 59 (8.4) | 0.8 |
| Central hypothyroidism, n (%) | 284 (30.5) | 222 (30) | 0.85 |
| Central hypoadrenalism, n (%) | 188 (20) | 157 (21.2) | 0.7 |
| Central hypogonadism, n (%) | 288 (31.3) | 242 (33.6) | 0.36 |
| GH at diagnosis, mean ± SD, μg/dL | 34.6 (68.8) | 34.8 ± 70 | 0.95 |
| IGF1 at diagnosis, mean ± SD, μg/dL | 755.5 ± 339.7 | 760.5 ± 338 | 0.72 |
| IGF1 at diagnosis, mean ± SD, ULN | 3.83 ± 4.1 | 3.85 ± 4.2 | 0.83 |
| Number of deaths, n (%) | 193 (18.7) | 153 (18.9) | 0.9 |

GH, Growth Hormone; IGF1, Insulin Growth Factor I; SD, standard deviation; ULN, upper limits of normal.

**Supplementary Table 2.** Multivariate model for independent predictors of mortality (n = 564).

| **Variables** | **HR** | **95% CI** | **P value** |
| --- | --- | --- | --- |
| Gender | 1.01 | 0.65-1.57 | 0.97 |
| Age | 3.98 | 2.65-5.99 | **< 0.001** |
| IGF1 above ULN at last follow-up | 2.52 | 1.59-4.01 | **< 0.001** |
| Therapy  - Surgical and medical vs multimodal with RT  - Surgical vs multimodal with RT  - Medical vs multimodal with RT | 1.45  1.94  1.31 | 0.83-2.51  0.90-4.19  0.68-2.55 | 0.19  0.09  0.42 |
| Hypertension | 1.13 | 0.67-1.90 | 0.64 |

HR, Hazard Ratio; IGF1, Insulin Growth Factor I; multimodal therapy includes any combination of at least two types of treatment (medical, surgical or radiation, either radiotherapy or radiosurgery); ULN, upper limits of normal.
